# Exploring the Role of Genetic Diversity and Cultural Norms in COVID-19 Vulnerability: A Global Analysis

**DOI:** 10.3390/ijerph22111690

**Published:** 2025-11-08

**Authors:** Andrew Phiri

**Affiliations:** Department of Economics, Nelson Mandela University, Gqeberha 6001, South Africa; andrew.phiri@mandela.ac.za

**Keywords:** COVID-19, expected heterozygosity, disease prevalence, collectivism, individualism, deep roots

## Abstract

The COVID-19 disease outbreak is the deadliest viral pandemic our generation has experienced, and much uncertainty remains over the varying vulnerability of different populations to the virus. This study investigates whether long-term evolutionary processes, such as genetic diversity and culturally embedded behavioural norms, can help explain why countries experienced different levels of COVID-19 infections and mortalities. Using a sample of 133 countries, we find that populations with higher expected genetic heterozygosity and greater historical exposure to infectious diseases are associated with lower COVID-19 case and death rates. We reveal two distinct pathways through which these effects manifest. Firstly, populations that migrated further from the evolutionary origins of Homo sapiens in East Africa exhibit lower genetic heterozygosity, which, in turn, is linked to greater susceptibility to COVID-19. Secondly, regions with higher historical disease prevalence tend to develop collectivist cultural norms and behaviours that are shaped to reduce disease transmission, which appear to mitigate the spread of COVID-19. These findings suggest that differences in vulnerability are not random but rather deeply rooted in genetic and cultural evolution. The analysis remains robust after accounting for socioeconomic, geographical, and institutional controls. Our findings offer policymakers fresh perspectives by integrating genetic theory and sociocultural dynamics into contemporary public health strategies.

## 1. Introduction

‘*…humans who domesticated animals [fell] victim to the newly developed germs, but those humans evolved substantial resistance to new[er] disease…*’

[1]

The above quote, taken from the Chapter 11 of Jared Diamond’s (1997) [1] non-fictional Pulitzer Prize-winning book *Guns*, *Germs and Steel*, explains how many of the deadliest viral diseases in human history first appeared during the Neolithic transition, when societies shifted from hunting and gathering to farming. Central to this evolutionary change was the domestication of animals, which, while essential for food, labour, and transport, also introduced new zoonotic diseases to humans. As Diamond (1997:197) [1] observed, these diseases evolved into viral pathogens that would become ‘*the biggest killers of people*’, ultimately shaping patterns of human mortality and societal development. This perspective that disease resistance is an evolutionary outcome of historical human-pathogen interactions forms the conceptual starting point of our study.

We extend Diamond’s thesis [1] by questioning whether deep-rooted biological and cultural adaptations explain why some modern societies have been more vulnerable to viral outbreaks like COVID-19 than others. While Diamond focused on long-term biological responses to animal domestication [1], our study brings this evolutionary framework into the 21st century by examining how genetic diversity and culturally evolved behavioural norms may have influenced the global distribution of COVID-19 infections and deaths.

Despite the wealth of literature generated in the wake of the COVID-19 pandemic, most studies focus on the socioeconomic, environmental, and epidemiological vulnerabilities of different populations to the virus, such as healthcare infrastructure, government policy responses, climate/temperatures, demographic factors, or individual-level comorbidities [2,3,4,5,6,7,8,9,10,11,12,13,14,15,16]. Yet, these factors often fail to explain surprising cross-country patterns. For example, Sub-Saharan African and South Asian countries, which are regions with limited healthcare resources, have experienced significantly lower COVID-19 morbidity and mortality compared to more affluent nations. This paradox suggests that deeper structural forces may be driving people’s susceptibility to contracting and spreading the virus.

**Figure 1:**
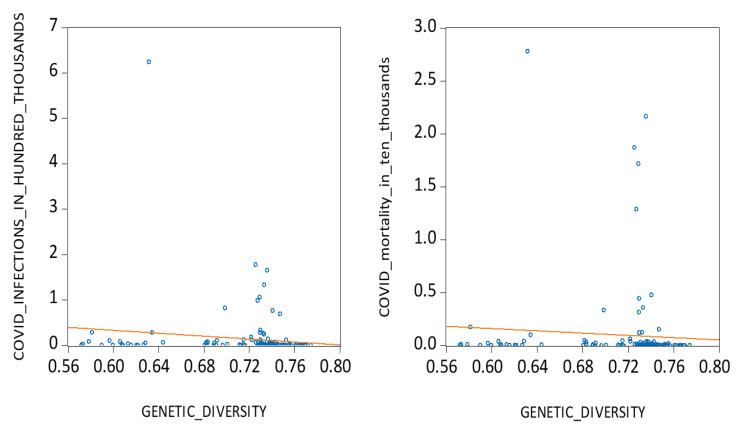
COVID infections, mortalities, and genetic diversity.

We address this unexplored terrain by proposing two complementary evolutionary explanations:

Firstly, from a genetic perspective, we argue that populations with higher genetic heterozygosity, which is an indicator of immune system diversity, are better equipped to resist novel viral pathogens [17,18]. According to the ‘Out of Africa’ migration model, populations that migrated farther from mankind’s origin in East Africa exhibit lower genetic diversity since they only carried a subset of genetic material when they migrated away from their parental colonies [19,20,21,22]. This may help explain why genetically homogeneous populations, particularly in the Americas and parts of Europe, were more severely impacted by COVID-19. To test this hypothesis, we examine the relationship between national levels of heterozygosity and average daily COVID-19 cases and deaths across 133 countries. As shown in Figure 1, the data reveal a notable inverse relationship, motivating our first hypothesis:

**H1.**  *Genetic diversity is inversely related to COVID-19 infections.*

**Figure 2:**
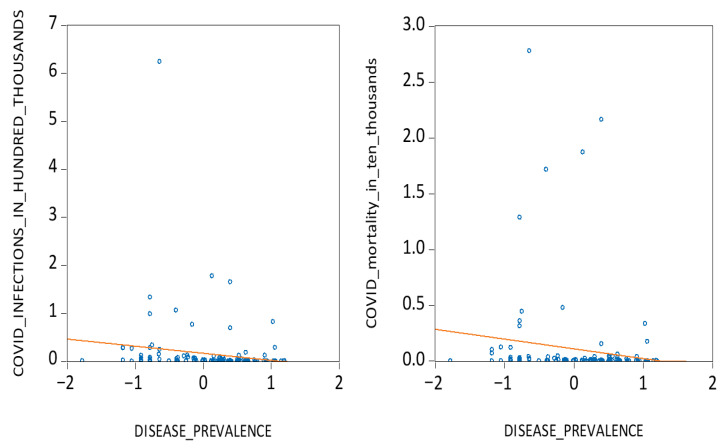
COVID infections, mortalities, and disease prevalence.

Secondly, from a cultural evolutionary standpoint, we draw on the pathogenic stress theory to explore how long-term exposure to disease shaped behavioural norms that mitigate viral transmission [23,24,25,26,27]. Societies historically exposed to high disease loads developed collectivist cultures that emphasize in-group loyalty, social compliance, and aversion to outsiders, which are traits that may foster greater adherence to public health guidelines such as masking and social distancing [28,29,30,31,32,33,34]. Using data on historical pathogen prevalence [26,27], we examine whether such legacies correlate with COVID-19 outcomes. As illustrated in Figure 2, countries with higher historical disease prevalence tend to report fewer infections and deaths, supporting our second hypothesis:

**H2.**  *Disease prevalence is inversely related to COVID-19 infections.*

We use the two-stage least squares (2SLS) method in our analysis because our key variables, genetic diversity and historical pathogen prevalence, may be correlated with unobserved factors that also influence COVID-19 outcomes, leading to biased estimates in ordinary regressions. To address this, we use instrumental variables that are theoretically strong predictors of our key variables but do not directly affect COVID-19 cases or deaths. For genetic diversity, we follow Ashraf and Galor [19] and use migratory distance from Addis Ababa, the origin of modern humans, which predicts variation in genetic diversity across populations but is unrelated to contemporary pandemic dynamics. For pathogen prevalence, we used Hofstede’s [31] cultural measures of collectivism, as used by Nikolaev and Salahodjaev [33] and Ang [28], since these cultural traits historically evolved in response to disease threats but are unlikely to directly cause COVID-19 infections today. These choices help us isolate the causal impact of our variables and are rooted in established evolutionary and cultural research, enhancing confidence in the instruments’ validity.

Overall, our study makes three main contributions. Firstly, it reframes COVID-19 vulnerability through the lens of long-run evolutionary forces, offering a novel theoretical framework. Secondly, it provides the first empirical test of the joint role of genetic diversity and culturally ingrained disease avoidance behaviours in explaining COVID-19 outcomes. Lastly, it highlights the importance of evolutionary-informed public health policies, suggesting that pandemic mitigation strategies may benefit from tailoring to the sociobiological profiles of different populations.

We structure the rest of the study as follows. The next section outlines the empirical regressions used in our study and describes the data used in our empirical analysis. Section 3 presents the baseline ordinary least squares (OLS) estimates of our regressions. Section 4 presents a two-stage least squares (2SLS) analysis to address potential endogeneity in the regressions. Section 5 presents further discussions of our results, whilst Section 6 concludes the study.

## 2. Methods and Data

Consistent with the two hypotheses specified in the study’s introduction, we estimate two cross-country least-squares regressions. Firstly, we model coronavirus infections/mortalities (SARS-CoV-2) as being endogenous to genetic diversity (GEN_DIV) and other conditioning variables (CONTROLS):

*SARS-CoV-2_i_* = μ + β *GEN_DIV*_i_ + *CONTROLS*_i_ + *ε*_i_ (1)

Secondly, we model coronavirus infections/mortalities (SARS-CoV-2) as being endogenous to pathogen prevalence (HPPI) and other control variables:

*SARS-CoV-2_i_ =* μ + β *HPPI + CONTROLS*_i_
*+ e_t_*_,*i*_ (2)

The dependent variable in Equations (1) and (2) is measured by total morbidities and total mortalities of the SARS-CoV-2 virus in 133 developing and developed economies sourced from the Johns Hopkins database (https://coronavirus.jhu.edu/region) and consists of total infections and mortalities as of 1 January 2022. The list of countries used in our study is provided in the paper’s Appendix A. The two main independent variables, GEN_DIV and HPPI, represent genetic diversity and disease prevalence, respectively, and, consistent with our formulated hypotheses, we expect a negative and statistically significant coefficient estimate on the β parameter, i.e., β < 0. As mentioned in the introduction, we use measures of expected heterozygosity from Ashraf and Galor [19] to capture genetic diversity, whereas we employ the 9-digit historical pathogen prevalence index found in Murray and Schaller [26] and Murray et al. [27] to measure disease prevalence.

We include three main sets of control variables in our analysis. Firstly, are geographic controls such as land suitability for farming (*land_suit*) collected from Michalopoulos [35], average elevation (*elevation*), geographic latitude (*latitude*), geographical longitude (*longitude*) collected from the CIA World Factbook, and distance to river collected from Gallup et al. [36]. Secondly, we use ecological controls including ecological fractionization (*eco_frac*) and ecological polarization (*eco_polar*), which are collected from Fenske [37], who measures ecological diversity by vegetation types. Thirdly, we use average temperature (*temp*) and average precipitation (*precip*) as climate controls, which we source from Harris et al. [38].

For our sensitivity analysis, we include five more sets of controls. Firstly, there are historical state development variables like the timing of the Neolithic transition (*Neolithic*) and state history (*Stat_Hist*) variables sourced from Putterman and Weil [39], as well as the timing of early human settlement (*Origtime*) sourced from Ahlerup and Olsson [40]. Secondly, we use the 2019 per capita level of health expenditure as a measure of the quality of modern health institutions (*health*). Thirdly, we employ the legal origins dummies of La Porta et al. [41], which categorize countries according to 5 legal systems (i.e., English, French, German, Scandinavian, or Socialist). Fourthly, we use the natural resources dummy (oil and gas reserve dummy), sourced from Lujala et al. [42]. Lastly, we use the dummy variables for Islands from the CIA World Factbook. We consider this later dummy important since most countries which have recorded the lowest cases of coronavirus are, incidentally, Island economies.

We also employ two-stage least squares (2SLS) estimators to address the potential heterogeneity problem of endogeneity within the regressions. To this end, we employ two sets of instruments. For the first set of instruments, we follow Ashraf and Galor [19] and use the migratory distance from Addis Ababa as an instrument for expected heterozygosity, which we then use to estimate COVID-19 infections and mortalities. For the second set of instruments, we follow Nikolaev and Salahodjaev [33] and Ang [28] and employ Hofstede’s measures of collectivism/individualism [31] as an instrumental variable for historical pathogen prevalence. The descriptive statistics of all the variables used in our study are summarized in Table A1 in Appendix B of the paper.

## 3. Baseline Analysis

### 3.1. OLS Estimates

This section of the paper presents the OLS estimates of the regressions (1) and (2), which are summarized in Table 1. Panel A reports the results between genetic diversity and COVID-19 infections/mortalities (i.e., columns (1)–(8)) whilst Panel B reports the results between disease prevalence and COVID-19 infections/mortalities (i.e., columns (9)–(16)). Columns (1), (5), (9), and (13) present the models without any control variables, which produce negative and statistically significant estimates at all levels of significance. Generally, these findings support Lively’s [8] hypothesis that populations with higher genetic diversity and more disease prevalence are associated with lower COVID-19 infections and mortalities. We are, however, concerned by the low R-squared values, particularly when genetic diversity is the independent variable (columns (9)–(16)) and the variable explains only between 2 and 6 percent of variation in COVID-19 morbidities and mortalities.

Suspecting omitted variables bias as the reason for these low explanatory power in the regressions, columns (2), (6), (10) and (14) present the models inclusive of four geographic controls (i.e., land suitability, average elevation, longitude and latitude) which significantly improves on all R-squared values which now explain between 28 and 48 percent of variation in the regressions. Moreover, we observe that geographic factors such as latitude and longitude are positively correlated with COVID infections and mortalities, whilst land suitability is inversely correlated with the disease. These findings imply that countries further from the equator and the prime meridian, as well as those with less suitable cultivation land, experience more COVID infections and mortalities.

In columns (3), (7), (11), and (15), we add two ecological controls (i.e., ecological fractionalization and polarization) whilst in columns (4), (8), (12), and (16), we add another two climate control variables (average temperature and precipitation). Notably, the addition of the last two sets of controls does not offer much change in the magnitude of regression estimates for genetic diversity and disease prevalence, and neither does it significantly improve the R-squared variable. We do, however, observe negative, statistically significant estimates for the temperature variable, indicating that areas with higher temperatures have lower COVID infections and deaths. Similar findings have recently been reported by O’Reilly et al. [43], who observed a low survival rate of the SARS-CoV virus in geographical areas with higher temperatures and humidity levels. Nevertheless, we note that across all regressions, the magnitude of the coefficient estimates on genetic diversity and disease prevalence variables is larger in absolute terms in comparison to coefficient estimates on other control variables, hence highlighting the dominance of evolutionary and behavioural factors in explaining movements in COVID-19 infections and mortalities.

### 3.2. Sensitivity Analysis: Additional Controls and Dummy Variables

As a robustness exercise, we re-estimate the baseline regressions after including more controls and dummy variables. Firstly, we include 3 sets of dummies for Islands, legal origins, and natural resources, and report these findings in columns (1), (5), (9), and (13) of Table 2. Secondly, in columns (2), (6), (10), and (14), we add controls for the number of years since the Neolithic transition from hunting and gathering to agricultural societies. Thirdly, in columns (3), (7), (11), and (15), we add controls for state history, which accounts for the depth of experience with state institutions above tribal levels within a territorial geographic scope. Fourthly, in columns (4), (8), (12), and (16), we add controls for the time elapsed since the original (uninterrupted) human settlement and for health expenditure as a proxy for the quality of current health institutions.

As can be collectively observed in Table 2, the inclusion of additional dummies and controls does not change the sign or the magnitude of the genetic diversity and disease prevalence variables, and yet we note an improvement in the explanatory power of all regressions (i.e., R^2^). We also note that none of the state and antiquity (S&A) variables (i.e., Neolithic transition, state history, and original time since human settlement) are significantly correlated with COVID-19 figures, implying that historical institutional advantages are non-detrimental towards COVID-19 infections and deaths. Moreover, the positive and significant coefficient estimate on current health institution variables reported in columns (4) and (16) further implies that economies with less advanced health institutions have been less affected by the COVID pandemic. This finding can be treated as additional evidence of factors other than technological and institutional factors being responsible for the differing patterns in the distribution of COVID-19.

## 4. Two-Stage Least Squares (2SLS) Estimates

So far, we have not addressed endogeneity in the estimated regressions. In this section of the paper, we use a two-staged least squares (2SLS) model in which we use instrument variables for genetic diversity and disease prevalence. In applying the 2SLS estimators to examine the relationship between genetic diversity and COVID-19 infections/mortalities, we specify the first stage regression as the one used in Ashraf and Galor [19,22,44]:

*GEN_DIV_i_* = μ + β *MIGR_DIST*_i_ + *CONTROLS*_i_ + *ε*_i_ (3)

And then extract the estimated values of genetic diversity from regression (3) and use them in the following second-stage regression:

*SARS-CoV-2_i_* = μ + β *GEN_DIV*_i_ + *CONTROLS*_i_ + *ε*_i_ (4)

Moreover, we follow Nikolaev and Salahodjaev [33] and Ang [28] who use disease prevalence as an instrument for collectivism/individualism measures of psychological behaviour proposed by Gelfand et al. [30]. In applying these instruments in our study, we propose the following 2SLS estimation regressions. Under the first stage regression, we model individualism/collectivism as being endogenous to disease prevalence:

*IND_COLL_i_* = μ + β *HPPI*_i_ + *CONTROLS*_i_ + *ε*_i_ (5)

And then we extract the estimates of individualism/collectivism and model them as being exogenous towards COVID-19 infections and mortalities in the second-stage regression:

*SARS-CoV-2_i_* = μ + β *IND_COLL*_i_ + *CONTROLS*_i_ + *ε*_i_ (6)

From regressions (3)–(6), we employ a set of controls for geography, climate, ecology, Islands, natural resources, and institutions as used in the previous section of the paper. Table 3 presents a summary of the 2SLS results, with Panel A reporting the estimates of regressions (3) and (4), whereas Panel B reports the estimates of regressions (5) and (6). Note that, as in the previous section of the paper, we present the estimates of our models in a stepwise fashion with columns (1), (5), (9) and (13) only including baseline controls and dummies (i.e., geography controls, climate controls, ecological controls, Islands dummies and natural resource dummies); columns (2), (6), 10) and (14) adding the number of years since the Neolithic transition; columns (3), (7), (11) and (15) adding State history; and lastly columns (4), (8), (12) and (15) adding time since original human settlement.

Based on the results reported in Panel A, we find that migratory distance is negatively and significantly related with genetic diversity in all first-stage regression estimators corresponding to columns (9)–(16) and we note that these findings are in alignment with those reported in Ashraf and Galor [19], Sequeira et al. [45] and Sequeira and Santos [44]. From the second-stage estimates reported in columns (1) to (8), we observe familiar negative and statistically significant estimates on the ‘instrumented’ genetic diversity variable. These findings confirm a mechanism in which longer (shorter) migratory distance from the origin is negatively (positively) correlated with genetic diversity, which then becomes a positive (negative) predictor of COVID-19 infections and mortalities. On the other hand, the first stage estimates from Panel B reveal a negative and statistically significant estimate between disease prevalence and collectivism as previously found in Nikolaev and Salahodjaev [27] and Ang [28], whereas the second stage estimates further reveal a negative correlation between the ‘instrumented’ collectivism variable and COVID-19 infections and mortalities. These findings confirm a mechanism in which populations with longer (shorter) historical exposure to pathogen diseases are associated with societies characterized by more (less) collectivist behaviour, which, in turn, is a negative (positive) predictor of COVID-19 infections and mortalities. This latter mechanism reflects the mechanism described in Chapter 5 of Diamond’s [1] book, which suggests that populations with longer experiences with diseases tend to “*[evolve] substantial resistance to new[er] disease*” ([1], p. 92).

## 5. Further Discussion of Results

### 5.1. Summary of Results

The findings of this study add a new layer to our understanding of COVID-19 outcomes by showing that deep-rooted biological and evolutionary traits, like genetic diversity and historical pathogen exposure, are powerful predictors of pandemic performance across countries. While earlier research has examined a wide range of more immediate determinants, such as public health responses [13], healthcare system readiness [7], population density and mobility [2,11], environmental conditions [4], and social vulnerabilities [9,12], this study steps back and asks a more fundamental question: Why are societies predisposed to behave in certain ways when facing a global health shock? By linking COVID-19 resilience to long-term evolutionary and cultural factors, as shaped by historical disease burden or genetic diversity, this research helps explain why even countries with similar policy responses sometimes experienced very different outcomes.

Importantly, this study does not reject the role of institutional or socio-economic variables, which are highlighted in the work of Acemoglu et al. [46] and many others, but rather suggests that these factors may operate alongside or even be shaped by deeper biological and behavioural patterns. This complements recent studies that look into genetic and immunological susceptibility to severe COVID-19 [15] or the impact of comorbidities [14], by showing that population-level traits such as collectivism or in-group orientation, rooted in pathogen-rich histories, may be just as important as individual-level health risks. The broader implication is that pandemic preparedness must go beyond institutional capacity or medical readiness to include an understanding of the historical and cultural fabrics of societies. These results therefore offer a bridge between the biological and the behavioural, the ancient and the modern, in explaining the complex geography of COVID-19’s global impact.

### 5.2. Managerial and Policy Implications

The findings of this study suggest that effective pandemic management requires more than just short-term interventions; it also demands a deep understanding of the long-term, structural traits of different societies. For policymakers, this means tailoring health strategies not just to the immediate needs of a population, but also to its cultural, behavioural, and even genetic predispositions. For example, countries with histories of high pathogen exposure may have developed more collectivist cultures that naturally support compliance with public health measures. In contrast, societies shaped by lower pathogen stress may require more deliberate communication strategies to achieve public cooperation. Recognizing these differences can help governments design more targeted, culturally sensitive responses that increase public trust and effectiveness.

For international organizations and global health managers, the results emphasize the importance of building resilience not only through infrastructure or medical supplies but also through long-term investments in public behaviour, education, and cultural understanding. Efforts to strengthen global pandemic preparedness should account for how historical experiences with disease shape today’s attitudes toward risk, cooperation, and authority. This means that what works in one country may not work in another, not because of technical failure, but because of deep-rooted behavioural patterns. Ultimately, this study calls for a more integrated approach to public health policy that acknowledges the invisible but powerful legacy of our evolutionary and cultural past.

### 5.3. Avenues for Future Research

While this study offers new insights into the deep-rooted drivers of pandemic outcomes, it is not without limitations. One key constraint is the use of cross-country data, which may mask important variations within countries, especially in large, diverse nations. Furthermore, some of the variables used, such as cultural or genetic traits, are proxies that may not fully capture the complexity of human behaviour or biological diversity. There is also the challenge of separating correlation from causation, particularly when dealing with historical or evolutionary influences that unfold over long time horizons. These factors mean that while the patterns observed are compelling, they should be interpreted with appropriate caution.

Future research can build on this work by exploring more granular data, including within-country or city-level variation in pandemic responses. Longitudinal studies could also help unpack how deep determinants interact with modern institutions, technology, and global mobility. Furthermore, interdisciplinary collaboration by economists, epidemiologists, geneticists, and anthropologists could deepen our understanding of how historical legacies shape real-time crisis outcomes. Exploring the role of communication, misinformation, and public trust as mediators between deep traits and pandemic behaviour could also yield valuable policy insights. Overall, our study opens a rich frontier for future work that bridges the past and present to better prepare for global health shocks.

## 6. Conclusions

This study highlights the powerful role of deep-rooted historical, genetic, and cultural factors in shaping how societies experienced the COVID-19 pandemic. By linking deep-rooted determinants, such as ancestral diversity, institutional quality, and cultural traits, to cross-country differences in COVID-19 outcomes, we show that vulnerability to global health shocks is not only a matter of immediate response or healthcare capacity, but also of enduring legacies. These findings add a fresh dimension to the growing literature on the determinants of COVID-19, complementing existing work on economic, environmental, and demographic drivers.

While our results are subject to the usual limitations of macro-level analysis, they suggest that future preparedness must look beyond surface-level indicators. Building resilience requires a deeper understanding of the historical and structural foundations of societies. As pandemics continue to test global systems, recognizing these underlying patterns can help inform more adaptive and context-sensitive public health and policy responses. In this way, the past remains a powerful lens through which to understand and shape the future.

## Figures and Tables

**Table 1:** Baseline regressions.

	Dependent Variable: Log (SARS-CoV-2)							
**Panel A**	**Infections**				**Mortalities**			
**Independent variable**	**(1)** **No controls**	**(2)** **Add biogeography**	**(3)** **Add ecology**	**(4)** **Add** **Climate**	**(5)** **No controls**	**(6)** **Add biogeography**	**(7)** **Add ecology**	**(8)** **Add** **Climate**
Gen_div	−12.91 (4.18) ***	−28.13 (4.17) ***	−26.52 (4.20) ***	−26.90 (4.52) ***	−8.33 (4.15) ***	−25.68 (4.73) ***	−24.46 (4.67) ***	−23.99 (5.03) ***
Land_suitability		−0.02 (0.008) ***	−0.12 (0.06) *	−0.13 (0.06) **		−0.02 (0.01)	−0.11 (0.07)	−0.11 (0.07)
Elevation		0.0004 (0.0005)	0.0002 (0.0006)	0.00003 (0.0006)		0.001 (0.0005) ***	0.0009 (0.0005) *	0.0009 (0.0005) *
Latitude		0.07 (0.01) ***	0.07 (0.01) ***	0.05 (0.01) **		0.07 (0.01) ***	0.07 (0.01) ***	0.06 (0.01) ***
Longitude		0.007 (0.004) *	0.007 (0.004) *	0.008 (0.004) **		0.007 (0.004) *	0.007 (0.004) *	0.007 (0.004) *
Dist_river		−0.0005 (0.0004)	−0.0005 (0.0004)	−0.0008 (0.0004)*		−0.0007 (0.0005)	−0.0008 (0.0005)	−0.0009 (0.0006)
Eco_frac			2.03 (1.25)	1.77 (1.25)			2.04 (1.46)	1.88 (1.53)
Eco_pol			−1.95 (1.31)	−1.43 (1.31)			−1.97 (1.29)	−1.81 (1.30)
Temp				−0.05 (0.03) *				−0.02 (0.03)
Precipitation_mean				−0.0005 (0.0003)				−0.0001 (0.0003)
Constant	15.97 (2.96) ***	25.39 (2.88) ***	24.54 (2.93) ***	26.56 (2.99) ***	9.68 (2.97) ***	20.15 (3.28) ***	19.56 (3.33) ***	19.83 (3.28) ***
R^2^	0.06	0.48	0.49	0.52	0.03	0.40	0.41	0.42
Obs	143	127	127	127	143	111	111	111
**Panel B**								
**independent variable**	**(9)** **No controls**	**(10)** **Add biogeography**	**(11)** **Add ecology**	**(12)** **Add** **Climate**	**(13)** **No controls**	**(14)** **Add biogeography**	**(15)** **Add ecology**	**(16)** **Add** **Climate**
Disease_9	−1.75 (0.29) ***	−1.19 (0.33) ***	−1.24 (0.29) ***	−1.24 (0.29) ***	−1.29 (0.35) ***	−0.81 (0.34) **	−0.82 (0.33) **	−0.83 (0.34)
Land_suitability		−0.05 (0.0) ***	−0.22 (0.07) ***	−0.17 (0.040) ***		−0.05 (0.01) ***	−0.19 (0.08) **	−0.13 (0.05) ***
Elevation		0.0003 (0.0006)	0.00005 (0.0007)	−0.0002 (0.0007)		0.001 (0.0005) **	0.0008 (0.0006)	0.0007 (0.0006)
Latitude		0.03 (0.01) ***	0.04 (0.01) ***	0.04 (0.009) ***		0.03 (0.01) ***	0.04 (0.01) ***	0.04 (0.01) ***
Longitude		−0.003 (0.004)	−0.002 (0.003)	−0.001 (0.003)		−0.003 (0.004)	−0.002 (0.003)	−0.001 (0.003)
Dist_river		−0.0007 (0.0005)	−0.0007 (0.0004) *	−0.001 (0.0005) *		−0.0009 (0.0005) *	−0.001 (0.0005) *	−0.001 (0.0006) *
Eco_frac			3.83 (1.47) **	5.41 (1.58) ***			3.22 (1.73) *	5.12 (1.82) ***
Eco_pol			−2.77 (1.40) *	−3.61 (1.56) **			−2.29 (1.51)	−3.67 (1.62) **
Temp				−0.01 (0.003) ***				−0.01 (0.003) ***
Precipitation_mean				−0.002 (0.003)				0.0001 (0.003)
Constant	6.99 (0.39) ***	6.54 (0.45) ***	6.46 (0.76) ***	6.55 (0.80) ***	3.81 (0.47) ***	3.00 (0.48) ***	2.86 (0.86) ***	2.74 (0.86) ***
R^2^	0.20	0.34	0.39	0.42	0.12	0.28	0.30	0.34
Obs	143	127	127	127	124	111	111	111

Notes: “***”, “**”, “*” denote 10%, 5% and 1% critical levels, respectively. White heteroscedasticity-consistent standard errors reported in ().

**Table 2:** Regressions inclusive of additional controls and dummies.

	Dependent Variable: Log (SARS-CoV-2)							
	Infections				Mortalities			
**Independent variable**	**(1)** **Add dummies**	**(2)** **Add** **Neolithic**	**(3)** **Add statehist**	**(4)** **Add** **Origtime + health**	**(5)** **Add dummies**	**(6)** **Add** **Neolithic**	**(7)** **Add statehist**	**(8)** **Add** **Origtime + health**
Gen_div	−26.97 (4.37) ***	−27.39 (4.64) ***	−27.36 (4.71) ***	−14.57 (5.93) ***	−25.21 (5.39) ***	−25.38 (5.56) ***	−25.35 (5.60) ***	−16.41 (7.69) **
Neolithic		0.28 (0.37)	0.27 (0.37)	0.43 (0.34)		0.16 (0.39)	0.15 (0.41)	0.19 (0.41)
Statehist			0.01 (0.26)	0.03 (0.20)			0.03 (0.27)	0.10 (0.28)
Origtime				−0.20 (0.18)				−0.21 (0.29)
Health				4.11 (1.09) ***				2.21 (1.34)
Ecology controls	Yes	Yes	Yes	Yes	Yes	Yes	Yes	Yes
Geography controls	Yes	Yes	Yes	Yes	Yes	Yes	Yes	Yes
Climate controls	Yes	Yes	Yes	Yes	Yes	Yes	Yes	Yes
Legal origin dummies	Yes	Yes	Yes	Yes	Yes	Yes	Yes	Yes
Island dummies	Yes	Yes	Yes	Yes	Yes	Yes	Yes	Yes
Natural resources dummy	Yes	Yes	Yes	Yes	Yes	Yes	Yes	Yes
Constant	22.53 (3.23) ***	20.45 (4.70) ***	20.50 (4.74) ***	9.85 (5.54) *	16.59 (3.61) ***	16.29 (4.38) ***	16.41 (4.47) ***	10.49 (5.58) *
R^2^	0.62	0.61	0.61	0.66	0.57	0.58	0.58	0.60
Obs	127	117	117	115	111	105	105	103
**independent variable**	**(9)** **Add dummies**	**(10)** **Add** **Neolithic**	**(11)** **Add statehist**	**(12)** **Add** **Origtime**	**(13)** **Add dummies**	**(14)** **Add** **Neolithic**	**(15)** **Add statehist**	**(16)** **Add** **health**
Disease_9	−1.51 (0.39) ***	−1.83 (0.44) ***	−1.83 (0.44)	−1.27 (0.67) *	−1.25 (0.46) ***	−1.54 (0.49) ***	−1.56 (0.48) ***	−1.08 (0.54) **
Neolithic		0.93 (0.76)	0.82 (0.67)	0.84 (0.60)		0.50 (0.39)	0.40 (0.40)	0.35 (0.37)
Statehist			0.29 (0.26)	0.27 (0.24)			0.27 (0.31)	0.27 (0.30)
Origtime				−0.51 (0.33)				
Health								3.23 (1.27) **
Ecology controls	Yes	Yes	Yes	Yes	Yes	Yes	Yes	Yes
Geography controls	Yes	Yes	Yes	Yes	Yes	Yes	Yes	Yes
Climate controls	Yes	Yes	Yes	Yes	Yes	Yes	Yes	Yes
Legal origin dummies	Yes	Yes	Yes	Yes	Yes	Yes	Yes	Yes
Island dummies	Yes	Yes	Yes	Yes	Yes	Yes	Yes	Yes
Natural resources dummy								
Constant	3.50 (1.26) ***	3.49 (4.15)	2.13 (4.36)	2.59 (5.26)	1.09 (1.45)	3.83 (3.31)	2.62 (3.51)	4.12 (3.04)
R^2^	0.58	0.58	0.58	0.60	0.52	0.55	0.55	0.58
Obs	127	117	117	117	111	105	105	103

Notes: “***”, “**”, “*” denote 10%, 5% and 1% critical levels, respectively. White heteroscedasticity-consistent standard errors reported in ().

**Table 3:** 2SLS estimates.

**Panel A:**	**Dependent variable: Log (SARS-CoV-2)**							
**2nd stage estimates**	**Infections**				**Mortalities**			
	**(1)** **Add dummies**	**(2)** **Add** **Neolithic**	**(3)** **Add statehist**	**(4)** **Add** **Origtime+health**	**(5)** **Add dummies**	**(6)** **Add** **Neolithic**	**(7)** **Add statehist**	**(8)** **Add** **Origtime+health**
Gen_div	−26.97 (4.37) ***	−27.39 (4.64) ***	−27.36 (4.71) ***	−14.57 (5.93) ***	−25.21 (5.39) ***	−25.38 (5.56) ***	−25.35 (5.60) ***	−16.41 (7.69) **
Neolithic		0.28 (0.37)	0.27 (0.37)	0.43 (0.34)		0.16 (0.39)	0.15 (0.41)	0.19 (0.41)
Statehist			0.01 (0.26)	0.03 (0.20)			0.03 (0.27)	0.10 (0.28)
Origtime				−0.20 (0.18)				−0.21 (0.29)
Health				4.11 (1.09)				2.21 (1.34)
Ecology controls	Yes	Yes	Yes	Yes	Yes	Yes	Yes	Yes
Geography controls	Yes	Yes	Yes	Yes	Yes	Yes	Yes	Yes
Island dummies	Yes	Yes	Yes	Yes	Yes	Yes	Yes	Yes
Natural resources dummy	Yes	Yes	Yes	Yes	Yes	Yes	Yes	Yes
Constant	22.53 (3.23) ***	20.45 (4.70) ***	20.50 (4.74) ***	9.85 (5.54) *	16.59 (3.61) ***	16.29 (4.38) ***	16.41 (4.47) ***	10.49 (5.58) *
R^2^	0.62	0.61	0.61	0.66	0.57	0.58	0.58	0.60
Obs	127	117	117	115	111	105	105	103
IV F-statistic	10.66 ***	8.52 ***	7.99 ***	8.77 ***	7.23 ***	6.63 ***	6.21 ***	5.75 ***
**1st stage estimates**	**Dependent variable: Log (Gen_div)**							
Migra_dist	−0.007 (0.00004) ***	−0.007 (0.00004) ***	−0.007 (0.00004) ***	−0.007 (0.00004) ***	−0.007 (0.00004) ***	−0.007 (0.00004) ***	−0.007 (0.00004) ***	−0.007 (0.00004) ***
Controls and dummies	✓	✓	✓	✓	✓	✓	✓	✓
R^2^	0.99	0.99	0.99	0.99	0.99	0.99	0.99	0.99
**Panel B:**								
**2nd stage estimates**								
	**(9)** **Add dummies**	**(10)** **Add** **Neolithic**	**(11)** **Add statehist**	**(12)** **Add** **Origtime+health**	**(13)** **Add dummies**	**(14)** **Add** **Neolithic**	**(15)** **Add statehist**	**(16)** **Add** **Origtime+health**
Coll_ind	−4.90 (2.23) **	−5.86 (2.63) **	−5.87 (2.73) **	−3.11 (1.75) *	−5.41 (2.66) **	−6.63 (2.93) **	−6.64 (3.04) **	−4.17 (2.15) *
Neolithic		2.95 (0.81) ***	2.99 (1.30) **	2.43 (0.95) **		3.53 (0.97) ***	3.60 (1.51) **	3.00 (1.32) **
Statehist			−0.05 (0.71)	0.41 (0.39)			−0.07 (0.80)	0.21 (0.55)
Origtime				−0.72 (0.39) *				−0.55 (0.48)
Health				2.73 (2.11)				2.18 (2.61)
Ecology controls	Yes	Yes	Yes	Yes	Yes	Yes	Yes	Yes
Geography controls	Yes	Yes	Yes	Yes	Yes	Yes	Yes	Yes
Island dummies	Yes	Yes	Yes	Yes	Yes	Yes	Yes	Yes
Natural resources dummy	No	No	No	Yes	No	No	No	Yes
Constant	9.16 (1.91) ***	16.24 (6.64) ***	16.65 (11.11)	7.96 (7.84)	5.54 (2.08) **	24.19 (7.94) ***	24.78 (12.92) *	17.15 (10.98)
R^2^	0.17	0.11	0.12	0.49	0.09	0.19	0.21	0.41
Obs	66	62	62	61	66	62	62	61
IV F-statistic	11.31 ***	16.66 ***	15.48 ***	20.73 ***	9.79 ***	16.76 ***	15.48 ***	20.93 ***
**1st stage estimates**	**Dependent variable: Log (Coll_ind)**							
Disease_9	0.68 (0.21) ***	0.72 (0.24) ***	0.70 (0.25) ***	0.55 (0.26) ***	0.68 (0.21) ***	0.72 (0.24) ***	0.70 (0.25) ***	0.55 (0.26) ***
Controls and dummies	✓	✓	✓	✓	✓	✓	✓	✓
R^2^	0.54	0.57	0.57	0.60	0.54	0.57	0.57	0.60
Obs	66	62	62	61	66	62	62	61

Notes: “***”, “**”, “*” denote 10%, 5% and 1% critical levels, respectively. White heteroscedasticity-consistent standard errors reported in ().

## Data Availability

The original data presented in the study are openly available from the Johns Hopkins database (https://coronavirus.jhu.edu/region (accessed on 1 January 2022)).

## Appendix A

List of countries: Afghanistan, Albania, Algeria, Angola, Argentina, Armenia, Australia, Austria, Azerbaijan, Belgium, Benin, Bolivia, Botswana, Brazil, Brunei, Bulgaria, Burkina Faso, Burundi, Cambodia, Cameroon, Canada, Central African Republic (CAR), Chad, Chile, China, Colombia, Congo, Costa Rica, Côte d’Ivore, Croatia, Czech Republic, Denmark, Djibouti, Democratic Republic of Congo, Ecuador, Egypt, El Salvador, Equatorial Guinea, Eritrea, Estonia, Eswatini, Ethiopia, Finland, France, Gabon, Gambia, Georgia, Germany, Ghana, Greece, Guatemala, Guinea, Guinea–Bissau, Hungry, Iceland, India, Indonesia, Iran, Iraq, Ireland, Israel, Italy, Japan, Joran, Kenya, Kuwait, Laos, Latvia, Lebanon, Lesotho, Liberia, Libya, Lithuania, Luxembourg, Macedonia, Madagascar, Malawi, Malaysia, Mali, Mauritania, Mexico, Morocco, Mozambique, Namibia, Nepal, Netherlands, New Zealand, Niger, Nigeria, Norway, Oman, Panama, Peru, Philippines, Poland, Portugal, Romania, Russia, Rwanda, South Korea, Saudi Arabia, Senegal, Serbia, Sierra Leone, Slovakia, Slovenia, Somalia, South Africa, Spain, Sri Lanka, Sudan, Suriname, Sweden, Switzerland, Syrian, Tanzania, Thailand, Togo, Tunisia, Turkey, United Arab Emirates, Uganda, United Kingdom, Ukraine, Uruguay, United States of America, Uzbekistan, Venezuela, Vietnam, Yemen, Zambia, Zimbabwe.

## Appendix B

**Table A1:** Summary statistics.

Variable	Source	Obs	Mean	s.d.	Minimum	Maximum
***Main dependent variables***						
SARS-CoV-2 (infections)	John Hopkins Institute	134	7.15	2.43	1.61	13.34
SARS-CoV-2 (mortalities)	John Hopkins Institute	134	3.62	2.58	0.00	10.23
***Main independent variables***						
Gen_div	Ashraf and Galor (2013) [19]	134	0.72	0.05	0.57	0.77
HPPI	Murray and Schaller (2010) [26]	134	0.29	0.67	−1.78	1.20
***Biogeography controls***						
Land suitability	Michalopoulos (2012) [35]	134	1.16	6.79	0.003	69.94
Average elevation	G-ECON project	128	562.18	475.11	0.64	2729.63
Latitude	CIA World Factbook	128	20.18	24.93	−42.00	64.00
Longitude	CIA World Factbook	128	19.90	51.98	−102.00	174.00
Distance to river	Gallup et al. (1999) [36]	134	305.05	380.60	15.00	2385.58
***Ecological controls***						
Ecological fractionalization	Fenske (2014) [37]	128	0.59	0.39	0.007	3.30
Ecological polarization	Fenske (2014) [37]	128	0.67	0.19	0.02	0.91
***Climate controls***						
Temperature volume	Harris et al. (2014) [38]	128	17.94	8.39	−4.94	28.55
Precipitation volume	Harris et al. (2014) [38]	128	992.08	706.75	21.49	2933.82
Legal origins dummy	La Porta et al. (1999) [41]	134	-	-	0	1
Island dummies	CIA’s World Factbook	134	-	-	0	1
Natural resource dummy	Lujala et al. (2007) [42]	129	-	-	0	1
Colony dummies	CIA’s World Factbook	129	-	-	0	1
***Historic institution controls***						
Years since Neolithic revolution (Neolithic)	Putterman and Weil (2010) [39]	118	10.50	16.39	5.99	145.60
State History (Statehist)	Putterman and Weil (2010) [39]	118	−0.77	1.29	−3.57	7.50
Duration of human settlement (Origtime)	Ahlerup and Olsson (2012) [40]	134	10.44	1.98	−2.51	11.98
2019 per capita expenditure on health	World Development Indicators	134	321,356.82	605.25	22.89	6086.3
***Instrumental variables***						
Migratory distance from origin (Dist_orig)	Ashraf and Galor (2013) [19]	118	7.29	1.82	0.00	11.81
Collectivism	Gelfand et al. (2004) [30]	52	5.18	0.76	3.53	6.37
